# *Lactobacillus* Strains for Vegetable Juice Fermentation—Quality and Health Aspects

**DOI:** 10.3390/biomedicines10112867

**Published:** 2022-11-09

**Authors:** Catalina Voaides, Oana Boiu-Sicuia, Florentina Israel-Roming, Medana Zamfir, Silvia Simona Grosu-Tudor, Iulia Roxana Angelescu, Calina Petruta Cornea

**Affiliations:** 1 Faculty of Biotechnologies, University of Agronomic Sciences and Veterinary Medicine of Bucharest, 59 Marasti Blvd., 011464 Bucharest, Romania; 2 Department of Microbiology, Institute of Biology Bucharest of the Romanian Academy, 296 Splaiul Independenţei, 060031 Bucharest, Romania

**Keywords:** lactic acid bacteria, fermented vegetable juices, functional beverages

## Abstract

Vegetable juices are new carrier variants for beneficial bacteria, representing an alternative to dairy-fermented products, especially for vegan, strict vegetarian, or allergic consumers. The aim of this study was to characterize several Romanian native lactic acid bacteria (LAB) strains to select valuable nutritional and probiotic strains for vegetable juice fermentation. Nineteen LAB strains were analyzed for antibiotic susceptibility (disc-diffusion method), the presence of antibiotic resistance genes, the presence of functional genes. and the production of organic acids by HPLC. Antibiotic resistant strains were observed only with ampicillin (Amp10) and kanamycin (K30), 79% and 32%, respectively, with results partially confirmed by molecular analysis. Multiplex PCR revealed the presence of *LBA1272*, *dltD*, *folP*, *agl*, *α-amy*, *malL*, and *ribA* genes, related to stress resistance, starch metabolism, and production of vitamins, except for *folK*. HPLC analyses were performed on beet roots (SF), tomato (TM), and a mixture of carrots, celery, and beet (MTS) juices. High values of lactic acid were recorded in all cases of LAB fermentation (5034–14,176 µg/mL). The maximum values recorded for acetic acid did not exceed 2.5 mg/mL having a positive influence on the product’s taste.

## 1. Introduction

Lactobacilli are beneficial bacteria generally regarded as safe for human and animal health [1,2] recognized and approved also by the European Food Safety Agency [3]. They offer various advantages as potential probiotics [4] and postbiotics [5]. In order to be used for food obtaining, microorganisms must not be cytotoxic, should not harbor any acquired antimicrobial resistance genes to clinically relevant antimicrobials or reveal any hemolysis activity; instead, they should bring benefits to their hosts.

Lactic acid bacteria play a very important role in the preservation and organoleptic profile of fermented food products, dairy or non-dairy, but at the same time, they are equally important in improving the composition and diversity of the intestinal microbiota of both humans and animals [4,6]. Among the most important beneficial effects of these bacteria are (1) the host’s immune system modulation through cell signaling; (2) the prevention of diarrhea caused by antibiotic-resistant microorganisms; (3) inflammatory bowel diseases treatment as well as irritable bowel syndrome treatment; (4) the improvement of lactose intolerance; (5) a decrease in cholesterol levels; (6) production of various inhibitory compounds, such as bacteriocins, organic acids, hydrogen peroxide, diacetyl, and carbon dioxide; (7) anti-diabetic action by reducing blood glucose level; (8) bio-preservation and shelf-life extension of perishable vegetables; and last but not least (9) prevention effects against life-threatening gastrointestinal infections through different biological mechanisms [7,8,9,10,11,12,13,14].

Vegetable juices are important components of the human diet, due to their high nutritiveness and health benefits, because they provide essential phytochemical compounds, such as organic acids, vitamins, minerals, oligosaccharides, polyphenols, dietary fibers, and other bioactive compounds with functional properties. Therefore, these vegetable juices represent a proper matrix for the fermentation process. Nowadays, functional vegetable drinks show an upward trend because they can incorporate beneficial microorganisms that stimulate human health and give a better taste. Microbial improved vegetable drinks serve as a friendly alternative for dairy foods, but present similar health-promoting properties, are cholesterol and lactose free, and also, satisfy vegan, vegetarian, or allergic consumers [4,7,15,16,17,18,19,20,21]. These advantages are based on the unhealthy effect of cholesterol contained in the fermented dairy products and, respectively, on the increasing number of lactose-intolerant or persons with allergies to cow milk proteins in the world. Moreover, this kind of vegetable processing through fermentation increases the added value of these products, promotes human health, and has an amazing market potential [22]. On the other hand, these fermented beverages represent an alternative to the large wastage of vegetables and fruits by increasing their shelf life, thus, being a sustainable solution [23].A detailed characterization of these LAB functions will optimize the selection process of the most valuable strains and significantly improve the application of probiotics to support human health. In this context, the aim of this study was to characterize several Romanian native LAB strains in order to select valuable probiotic strains for vegetable juice fermentation (beet juice, tomato juice, and a mixture of celery, carrots, and beet juice). Another objective of the study was to obtain “functional beverages” (microscale-controlled conditions) with increased quality for potential consumers. Strains were analyzed for their safety, while the fermented beverages were investigated from organic acid production, antibacterial potential, and a respective, functional properties point of view.

## 2. Materials and Methods

### 2.1. Biological Material

Nineteen strains of lactic acid bacteria belonging to the Microbiology Department of Institute of Biology Bucharest of the Romanian Academy were used in this study (Table 1). These LAB strains were previously isolated from various vegetable sources or traditionally fermented food products (bors, pickles, fermented cabbage juice) and were selected based on their functional properties, such as antibacterial/antifungal activity, bacteriocins, polysaccharides, and surfactants production, as well as probiotic potential [24,25,26,27,28,29,30,31,32,33,34].

LABs were grown in MRS broth (De Man, Rogosa, and Sharpe liquid medium) (Carl Roth GmbH + Co.KG, Karlsruhe, Germany) at 36 °C, except for the P109, P124, and 21.2 strains, which were grown at 30 °C. The ST111 strain was grown in M17 broth (HiMedia Laboratories Pvt. Ltd., Thane, Maharashtra, India) supplemented with 10% filter-sterilized lactose solution. Solid media were used for antagonism studies, and for that, bacteriological Agar (PanReac AppliChem, Dublin, Ireland) was added to the above-mentioned media.

### 2.2. Antibiotic Susceptibility

Antibiotic susceptibility was performed using the disc-diffusion method in 9 cm diameter Petri dishes. The test was performed on 20 mL MRS or 20 mL M17 agar, on which 100 µL of freshly grown LAB cultures were plated. Commercially available antibiotic discs were then placed on top of the inoculated media. Four discs were distributed to each plate, while eight different antibiotics of clinical interest were tested: ampicillin (AMP10), chloramphenicol (C30), erythromycin (E15), gentamicin (CN30), kanamycin (K30), rifampicin (RD30), streptomycin (S300), and tetracycline (TE30) (Table 2).

Plates were incubated for 1 to 2 days at the optimal temperature for the microbials to grow. Bacterial inhibition zones around the antibiotic discs were visually analyzed to appreciate the sensitivity or tolerance of the studied strains to antibiotics. Biometric evaluation was also performed. Antibiotic resistant strains were determined as ones where bacteria growth was not disturbed.

### 2.3. DNA Extraction

LABs DNA extraction was performed using the Omega E.Z.N.A^®^ Bacterial DNA kit (Omega Bio-tek, Inc., Norcross, Georgia 30071, USA) following the manufacturer’s instructions. For the molecular studies, an extra LAB strain of *Lactobacillus plantarum* LAB43 was included into analysis as a previously analyzed strain (reference strain). The DNA concentrations were determined by UV light absorbance measurements (at 260 nm), while the purity was determined by 260/280 nm ratio and 260/230 nm ratio using the SpectraMax^®^ QuickDrop™_._ Micro-Volume Spectrophotometer (Molecular Devices, LLC, San Jose, CA, USA).

### 2.4. PCR for Antibiotic Resistance Genes

Several primer pairs were used to detect the antibiotic resistance genes (Table 3) in multiplex or single PCR reactions. Multiplex PCR reactions were carried out with the KAPA2G Fast Multiplex PCR kit (KAPA Biosystems Pty (Ltd), Cape Town 7925, South Africa) with 25 µL total reaction volume (buffer 2×, each primer 10 µM, ultrapure water, DNA~50–100 ng). For the individual PCR reactions, DreamTaq green PCR master mix (Thermo Scientific™, Vilnius, Lithuania) was used for 20 µL total reaction volume (buffer 1×, ultrapure water, each primer 10 µM, DNA~50–100 ng). The amplification program consisted of an initial denaturation at 95 °C for 10 min, followed by 30 cycles of denaturation at 95 °C for 1 min, annealing for 1 min (Table 3), and elongation at 72 °C for 1 min, with a final elongation at 72 °C.

The antibiotic resistance genes analyzed in this experiment were: (a) genes encoding antibiotic modifying enzymes that confer resistance to aminoglycosides and chloramphenicol, such as *aac(6′)-aph(2″)*, *aph(3′)-IIIa*, *ant(6)*, and *catA* genes [35]; (b) genes encoding for ampicillin resistance, such as *bla* and *blaZ* genes [36]; and (c) genes encoding target modifying enzymes whose products modify ribosomal RNA so that the erythromycin and tetracycline cannot bind to the ribosome, such as *erm(B)-1* and *tet(M)* genes [37], as in Table 3.

The PCR amplification products were examined by 2% agarose gel electrophoresis in 0.5× TBE buffer, supplemented with ethidium bromide. The electrophoretic profiles were then analyzed under UV light using the BioDoc-It imaging system (Ultra-Violet Products Ltd., Upland, CA, USA). 

### 2.5. Multiplex PCR for Functional Genes

To emphasize the presence of functional genes in the studied LAB strains, several pairs of primers were used in multiplex PCR reactions (Table 4). Multiplex PCR reactions were performed with the KAPA2G Fast Multiplex PCR kit (KAPA Biosystems Pty (Ltd), Cape Town, South Africa): 25 µL total reaction volume containing 2× multiplex mix, 10 µM each primer, ultrapure water, and 50 to 100 ng DNA. The amplification program consisted of an initial denaturation at 95 °C, followed by 30 cycles of denaturation at 95 °C for 15 s, annealing for 30 s (Table 4), and elongation at 72 °C for 90 s, with a final elongation at 72 °C.

The PCR amplification products were examined by 2% agarose gel electrophoresis in 0.5× TBE buffer, supplemented with ethidium bromide. The electrophoretic profiles were then analyzed under UV light using the BioDoc-It imaging system (Ultra-Violet Products Ltd., Upland, CA, USA) 

### 2.6. Testing the Fermentation Qualities of Selected LAB Strains

Selected LAB strains were used for vegetable juice fermentation in microscale-controlled conditions. The used LAB strains were L58, L61, 26.1, CR1, and BR9, along with a mixture of these five strains in equal parts, which was the encoded LAB mix. Three types of vegetable juice were used in this study, all made from fresh legumes (Table 5).

The lactic ferments consisted of 10^10^ CFU/ml LAB cells suspension. The biomass was harvested from freshly grown LAB cultures in MRS broth, centrifuged at 6000× *g* for 10 min.

The fermentation was initiated by inoculating the juices with LAB suspension (10:1 *v/v*). Uninoculated control juices were also prepared. The process was carried out at 35 °C, in darkness, for 4 days, under microbiologically controlled conditions.

The fermented beverages were analyzed both organoleptically (50 volunteers divided into panels, using the hedonic test) and biochemically by HPLC method.

### 2.7. Analyzing the Juice for Antibacterial Activity

The fermented tomato juice was screened for antibacterial ability against 5 pathogenic strains: *Bacillus cereus* ATCC 11778, *Escherichia coli* ATCC 8739 and ATCC 11229, and *Staphylococcus aureus* ATCC 6538 and ATCC 33592. Fresh pathogenic cultures of 20 h obtained in Tryptic Soy Broth (TSB, Oxoid™, Basingstoke, UK) were included in Luria Bertani Agar (LB, Lennox recipe, Carl Roth GmbH + Co.KG, Karlsruhe, Germany) at 1:100 *v/v* ratio. On top of this substrate 10 µL of LAB fermented and uninoculated tomato juice were added in spots and the plates were incubated over night at 36 °C. The antibacterial activity was than evaluated biometrically by measuring the clear halos of pathogenic growth suppression.

### 2.8. Organic Acid Analysis by HPLC Method

The method used to determine lactic and acetic acids is based on their separation by high performance reverse phase chromatography (HPLC-RP), using a resin-based ion exclusion chromatographic material.

*Sample preparation*. The samples were centrifuged at 10,000 rpm for 10 min. One milliliter was harvested from the aliquot and diluted 1/10 with ultrapure water. Prior to injection, samples were filtered through 0.20 µm pore cellulose syringe filters and placed in brown glass HPLC vials.

*Separation.* The samples processed as mentioned before were placed in the autosampler and injected into the chromatographic system (WATERS system, ALLIANCE 2695 with UV detector 2487, Milford, MA, USA). The acquisition, processing, and reporting of data was performed with EMPOWER 2.0 software. For separation, a SUPELCOGEL H column (with spherical particles of 9 µm, the inner diameter of the column 4.6 mm, and the length of the column 25 cm) was used. The mobile phase was 0.1% phosphoric acid solution, the elution being made in an isocratic system with 0.17 mL/min flow. The sample injected volume was 10 µL, and the separation was performed at 30 °C for 50 min. The detection of the carboxylic acids was performed with a UV detector at 210 nm.

*Identification and quantification.* The identification of the acids was based on the retention time recorded for the standard solutions, 14.85 min for lactic acid and 17.38 min for acetic acid.

The quantification was performed compared to the calibration curves obtained by injecting different volumes of 1.2 µg/mL lactic acid and 2.06 µg/mL acetic acid standard solutions. The calibration curve obtained for lactic acid was r^2^ = 0.998766, and that for acetic acid was r^2^ = 0.999021.

The samples were prepared and analyzed in triplicate, and the results are expressed as mean ± SD (standard deviation). One-way analysis of variance (ANOVA) was used for determination of significant quantitative differences.

## 3. Results and Discussions

### 3.1. Susceptibility of LAB to Antibiotics

In food, antibiotic resistant LAB represents a risk factor for consumer health, due to potential transfer of antibiotic resistance genes to opportunistic human pathogens, thus, causing complications in the antibiotic treatment of the patients. To prevent such deleterious effects, it is mandatory to control all stages of the food production flow and to exclude those lactic ferments that represent a source of genetic material that can induce resistance traits. These preventive measures can suppress resistant pathogenic strain development and multidrug-resistant strains [1,22,36,37,39].

Within the present study, the 19 LAB strains were analyzed with respect to eight antibiotics using the disc-diffusion method. Biometric evaluation of LAB growth inhibition zones revealed their sensitivity (S) or tolerance (T) to each tested antibiotic (Table 6). Those strains revealing clear inhibition zones for at least 2 cm from the antibiotic discs were considered sensitive, while the other showing whack growth were considered tolerant to the tested antibiotic concentration with a high risk of developing antibiotic resistance over time. Resistant strains were considered those LAB having normal development in the presence of tested antibiotics.

Among the tested LAB strains, antibiotic resistant strains were observed only to ampicillin (Amp10) and kanamycin (K30) (Table 6).

**Figure 1:**
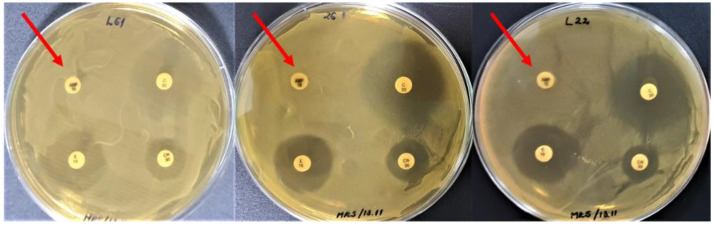
LAB strains revealing ampicillin resistance (arrows).

The LAB strains revealing antibiotic resistance to ampicillin (10 µg/disc) were: L22, 26.1, L61, L35, 19.3, 34.9, P40, P50, 21.2, P109, P124, Fv52, Fv177, ST111, and IBB (Figure 1), representing 79% of tested LAB. The L26, L58, BR9, and CR1 strains showed tolerance to this antibiotic concentration as they grew in low-density around the ampicillin disc (Amp10).

To chloramphenicol (30 µg/disc), L35, L58, 21.2 and P124 strains showed tolerance, developing low-density bacterial growth for 5 to 10 mm around the antibiotic discs. All the other strains (79% of tested LAB) were sensitive, revealing no bacterial growth for up to 11 mm around the antibiotic discs.

To gentamicin (30 µg/disc), more than 90% of the tested bacterial strains showed sensitivity, among which the L22 strain had the larger clear inhibition zone (15 mm) around the antibiotic disc. The Fv177 and L35 strains were revealed to be tolerant to this antibiotic. These results disagree with those from the scientific literature, which show a high resistance rate among lactobacilli (66.7%) to this antibiotic [35,39]. On the other hand, Zhou et al. [1] as well as Guo et al. [36] showed susceptibility to gentamicin in some LAB strains.

Regarding erythromycin (15 µg/disc), 74% of the analyzed LAB strains were sensitive, except for L58, P50, 21.2 ST111, and IBB strains, which were tolerant. Different variations in susceptibility to this antibiotic were also observed by Gad et al. [37]. Similar results were obtained in other scientific studies [36,37,40,41,42,43].

To kanamycin (30 µg/disc), six resistant LAB strains were observed, P40, P50, 21.2, P109, P124, and Fv177, all also having ampicillin resistance at 10 µg/disc. All the other bacterial strains, except for 19.3 and ST111, proved to be tolerant, developing low-density growth around the antibiotic discs. However, these two strains were completely inhibited to grow for 2 mm and 5 mm, respectively, around the kanamycin discs.

All 19 analyzed strains were found to be sensitive to moderately susceptible to rifampicin. However, the P109 strain was tolerant to the tested antibiotic concentration (30 µg/disc), developing a low-density growth for 5 mm around the discs.

To streptomycin (300 µg/disc), 53% of the LAB strains showed sensitivity (L22, L26, 19.3, 34.9, P40, Fv52, BR9, CR1, IBB, and ST111 strains), maintaining clear zones of complete growth inhibition around the antibiotic discs. In contrast, 21.2, 26.1, L35, L58, L61, P50, P109, P124, and Fv177 strains (47% of tested LAB) showed tolerance to this antibiotic concentration, developing low-density bacterial growth around the streptomycin discs. Similar results were obtained by Guo et al. [36]. On the other hand, there are other studies showing LAB resistance to streptomycin [1,35,37,39].

**Figure 2:**
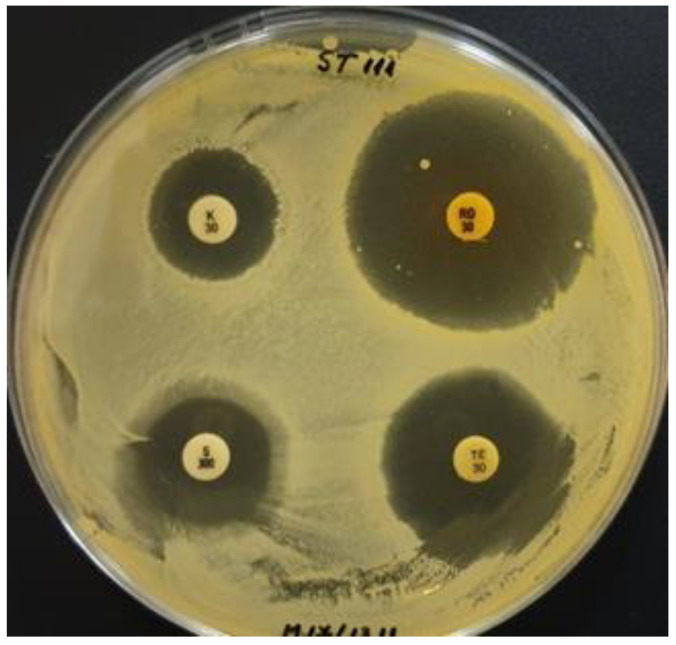
Susceptibility of ST111 strain to four antibiotics: kanamycin (K30) (left-up), rifampicin (RD30) (right-up), streptomycin (S300) (left-down), and tetracycline (TE30) (right-down).

Tetracycline (30 µg/disc) was able to completely inhibit 19.3, IBB, and ST111 growth (Figure 2). For these strains, clear inhibition zones of 8–9 mm around the antibiotic disc were observed.

In the case of the ST111 strain, it was noticed that around the streptomycin disc (S300), no growth was developed for 5 mm from the disc, the strain being sensitive to high concentrations of this antibiotic. However, as the antibiotic concentration diminished in the substrate due to the gradient diffusion, the ST111 strain was able to develop low-density growth on a 3 mm long area.

### 3.2. LAB Molecular Characterization

Only 13 of the studied LAB strains were used in molecular analysis (L22, L26, L35, L58, L61, BR9, CR1, Fv52, Fv177, P124, ST111, 26.1, and 34.9 strains) as they revealed better stability while sub-culturing. Together with those, the *Lactobacillus plantarum* LAB43 strain was used as reference.

#### 3.2.1. Antibiotic Resistance Genes

Multiplex PCR performed to emphasize the presence of antibiotic resistance genes partially confirmed the microbiological results on antibiotic susceptibility (Table 7). These uncorrelated facts can be explained by several mechanisms: (1) the LAB strains inactivate the antibiotic by destroying or modifying the drug itself so that it is no longer toxic for the cell (intrinsically resistance); (2) some mutations of the antibiotic target site lead to their impossibility to bind; (3) the resistance genes were incomplete to be detected through PCR reaction; (4) there may be other genes responsible for the expression of antibiotic resistance than those used for PCR reactions (transposon or plasmid-carried genes); (5) factors that influence the susceptibility/resistance to an antibiotic—the inoculum size, the incubation conditions (time, temperature), and the medium [1,37].

In some clinical cases (patients with antibiotic-induced diarrhea or antibiotic treatments), the use of resistant lactobacilli strains could be indicated in order to survive in those stressful conditions and to re-establish the normal functional gastrointestinal microbiota [39]. Conversely, for the food safety domain, the presence of antibiotic resistant lactobacilli strains is not allowed due to the possibility of transferring the resistance genes to different pathogenic microorganisms [39].

Chloramphenicol resistance genes were not identified in any of the analyzed strains, which confirms the phenotypic characters obtained, the strains being sensitive to this antibiotic. In the case of ampicillin resistance, two genes were searched for this characteristic. In five of the analyzed strains (26.1, 34.9, P124, Fv52, and ST111), the phenotypic results were confirmed, but amplification products were also obtained in strains that were phenotypically tolerant (L26, L58, and BR9). According to the literature, there are several mechanisms that influence this result [1,37]. For streptomycin, the amplification product was obtained only for the L58 strain, although this bacterial strain phenotypically showed tolerance. However, the lack of the amplification product in the other strains confirms the phenotypic character of the sensitive/tolerant strains. These results regarding the correlation between different ranges of susceptibility to streptomycin and the presence of the ant(6) gene are confirmed in other research articles [1,35,36,37]. Regarding the kanamycin, a specific amplicon was obtained only for a single strain that was phenotypically resistant (P124) and for three other strains that were phenotypically tolerant (26.1, BR9, and CR1).

#### 3.2.2. Functional Genes in LAB

Multiplex PCR analysis revealed in most of the LAB tested strains the presence of all studied functional genes related to stress resistance, starch metabolism, and vitamin production, except for *folK* (Table 8), even if the intensity of the band that corresponds to the amplicon was variable.

**Figure 3:**
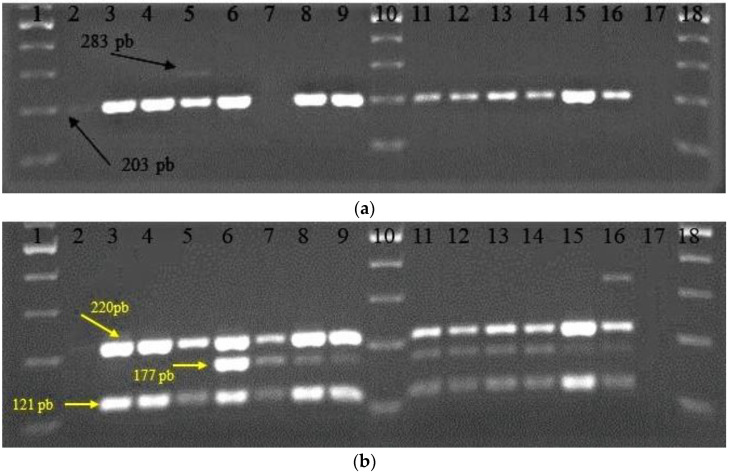
Electrophoretic pattern for multiplex PCR: (**a**) *LBA1272*, 203 pb (black arrow) and *dltD*, 283 pb (black arrow); (**b**) *α-amy*, 220 pb (yellow arrow); *malL*, 177 pb (yellow arrow); and *ribA*, 121 pb (yellow arrow). Samples: 1,10,18—ladder 100 pb, 2—L22, 3—L26, 4—L35, 5—LAB43, 6—L58, 7—L61, 8—BR9, 9—CR1, 11—Fv52, 12—Fv177, 13—P124, 14—ST111, 15—26.1, 16—34.9, 17—no DNA.

Among the studied functional genes in LAB, the *LBA1272* gene involved in bacterial survival to extreme pH conditions was determined for all bacterial strains, except for the L61 strain. The other gene (*dltD*), having similar functions, was only detected in the reference strain LAB43 (Figure 3a). Furthermore, it was observed that the L22 strain revealed only the *LBA1272* gene was involved in bacterial survival in extreme pH conditions.

When the presence of the genes for starch metabolism (*α-amy* gene responsible for amylase production) was analyzed, the corresponding amplicon was determined for all the studied LAB strains except the L22 strain. In the case of the *malL* gene (responsible for oligo-1,6-glucosidase production), also involved in starch metabolism, the corresponding amplicon was absent only for four strains (L22, L26, L35, and LAB43). Furthermore, the *ribA* gene involved in riboflavin synthesis was determined for all studied LAB strains, except for L22 (Figure 3b). There are several studies regarding genes from LAB strains involved in probiotic functions and nutrition [44,45,46,47,48].

### 3.3. Suppressive Effects of LAB Fermented Juice

**Figure 4:**
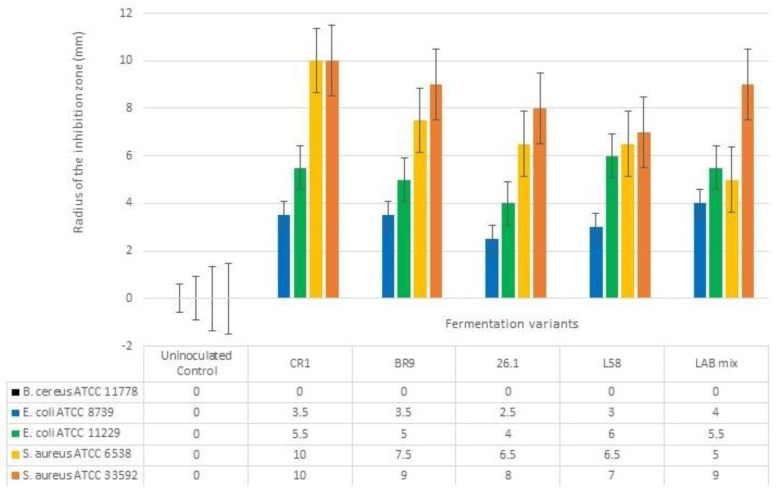
Antibacterial effect of fermented tomato juice. The error bars represent the standard error. Different letters indicate significant difference between fermented variants regarding their antibacterial activity against tested human pathogens.

The diffusion assay was tested to evaluate the antibacterial activity of fermented tomato juice against five pathogenic bacteria. If the pathogenic growth was completely inhibited, then clear halos were revealed surrounding the tomato juice spots, such cases suggesting the suppressive effect of tested tomato juices. However, if a blurry growth was seen in the inhibition halos, the pathogens were considered tolerant to the bioactive compound found in the juice. When unfermented tomato juice revealed no halos surrounding the inoculation spots, we considered the juice to lack antibacterial bioactive compounds. However, if no inhibition halos were seen in both fermented and unfermented tomato juice, we considered that pathogen as resistant to the bioactive compounds present in the juice. The antibacterial activity was biometrically evaluated by measuring the radius of the clear inhibition zones (Figure 4).

**Figure 5:**
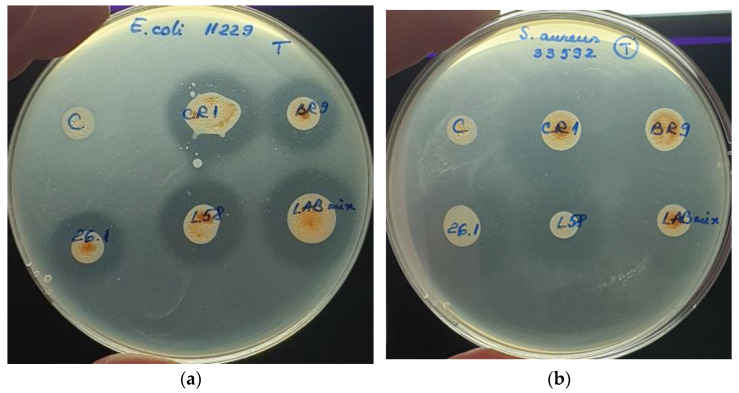
Antibacterial effect of fermented tomato juice: (**a**) *E. coli* ATCC 11229; (**b**) *S. aureus* ATCC 33592.

The fermented tomato juices revealed suppressive effect against both *E. coli* strains (Figure 5a). Inhibitory effects were also seen against *S.aureus* strains, even if these two pathogenic strains were not completely suppressed (Figure 5b). However, no antibacterial effects were seen when unfermented tomato juice was tested. Neither fermented variant was able to inhibit *B.cereus* growth.

Antibacterial activity of LAB is considered by other research groups to be performed by various metabolic compounds. However, the lactic acid and acetic acid had the highest inhibitory potential [49].

### 3.4. Organic Acids in LAB Fermented Vegetable Juices

HPLC analysis for lactic and acetic acid quantification were performed on the three vegetable juices, SF, MTS, and TM. The juices were fermented for 3 days with five LAB strains, CR1, BR9, 26.1, L58, and L61, as well as with a mixture of the five, and compared with uninoculated controls.

**Figure 6:**
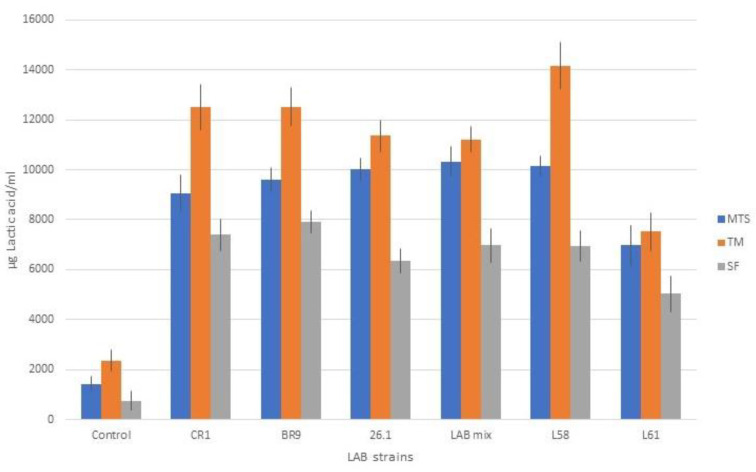
Lactic acid content of juice samples fermented with lactic acid bacteria.

The amount of lactic acid was influenced by both studied factors: the fermented juice (the substrate) and the LAB used (Figure 6). However, high values of lactic acid were recorded in all cases of LAB fermentation (from 5034 to 14176 µg/mL), compared to the uninoculated juices (from 744 to 2358 µg/mL).

Regarding the lactobacilli influence on lactic fermentation, it can be stated that they all led to significant increased amounts of lactic acid compared to the experimental control, in which no ferment was used.

Analyzing the data (Figure 6), strain L61 was less productive in terms of lactic acid, a fact also confirmed by the final pH values recorded in the juices fermented with this strain, values that were higher by approximately 0.5 units compared to the juices fermented with the other LAB strains.

In the case of tomato juice (TM) fermentation, even if the initial pH value of the fermentation medium was 4.5, the presence of high amounts of reducing mono-carbohydrates (13.02 ± 0.46 g glucose/L and 8.95 ± 0.38 g fructose/L in tomato juice [50]) significantly influenced the development of lactic bacteria and the synthesis of lactic acid (*p* < 0.05).

For the carrot, celery, and beet juice mix (MTS) and beet juice (SF), the lower content of free reducing mono-carbohydrates (2.62 ± 1.06 g glucose/L and 1.51 ± 0.85 fructose/L [51]) was compensated for by the higher initial pH value (6.0–6.5), which is in the optimal range for most lactic acid bacteria.

Compared to the TM and MTS juices, in beet juice (SF), the lactic acid content was lower (*p* < 0.05 when compared with TM and insignificantly when compared with MTS). However, between SF and MTS vegetable juices, the same trend line of lactic acid content was seen. Similar results were obtained also by other research groups. Thus, Rakin et al. [7] stated that lactic acid production is more intensive in fermented carrot juice. They consider that lactic acid influences the nutritive value, the taste, and the structure of the product. Another research group [52] is stating that the addition of carrot juice improved the fermentation and the production of lactic acid, increasing the mineral content in the resulting product.

**Figure 7:**
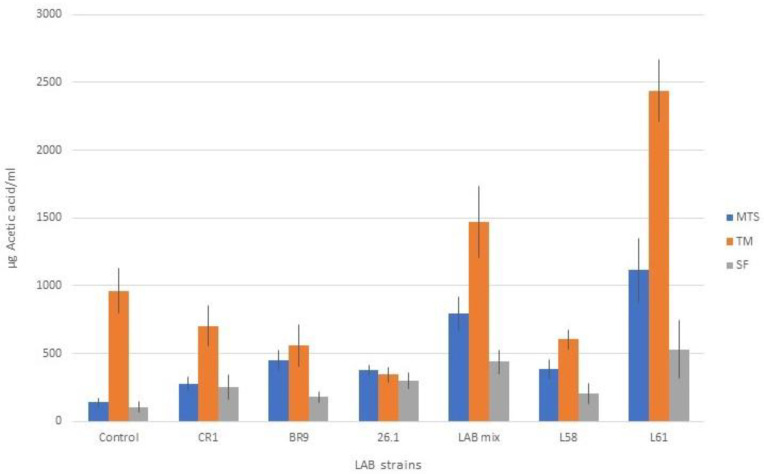
Acetic acid content in juice samples fermented with lactic acid bacteria.

The experimental data also emphasized the presence of acetic acid in the fermented samples but at a much lower level compared to lactic acid (*p* < 0.001). Similar to the lactic acid, acetic acid biosynthesis was influenced by both the substrate and the bacterial strains used for juice fermentation (Figure 7). Higher acetic acid values were obtained in TM and MTS fermented juices. Analyzing the data, the L61 strain led to significantly higher values of the determined analyte compared to the other bacterial strains. The presence of acetic acid in fermented juices is slightly disputed by the literature data [7,53]. Buruleanu et al. [54] found that the presence of ascorbic acid in vegetable juices produced by the probiotic lactobacilli strains also has nutritional importance and promotes anaerobic conditions (as an oxygen scavenger).

A reasonable level of acetic acid is beneficial for the sensorial qualities, giving a slightly acidic taste. Moreover, it protects the product’s shelf life. On the other hand, too large an amount of acetic acid can negatively influence the sensory qualities of the product through a strong acid taste and a typical pungent smell. We showed that the maximum values recorded in this experiment when using the selected fermenting LAB did not exceed 2500 µg/mL and had a positive influence on the taste.

All these characteristics of the studied selected LAB strains make them “helpful” bacteria. The interaction between the gut bacteria and the ingested “good” bacteria has several results: influence the immune activity, influence the digestion and the metabolism, and influence the response to the pathogen action, through strengthening the gut microflora [55]. Furthermore, these kind of LAB strains represent a cost effective, friendly, simple, and natural way of improving, helping, and sustaining the natural host gut microflora. The alternative is represented by the chemical drugs or chemical supplements. For instance, in the competition with the pathogens, the probiotic LAB strains have the ability to compete for the resources, for the site receptors and to stimulate the immune system of the host [55]. Therefore, the microorganisms or the consortia from the vegetables fermented beverages represent a healthy source of promoting components, a way to fight against pathogens and to support the natural gut microflora. In addition, vegetable fermentation will improve the bioavailability of the compounds (proteins, vitamins, fibers, phenols, amino acids, etc.) The organoleptic analysis of juice samples fermented with lactic acid bacteria, performed with volunteers divided into panels, using the hedonic test, led to some partial conclusions:

— in the case of MTS (carrot-celery-beet) juice, the fermentation with LAB mix gave the most balanced taste and aroma; such vegetable juice, when fermented, can be used as salad dressing
— in the case of SF (beet) juice, the most appreciated experimental variants were those fermented with L58 and 26.1, respectively. The respondents appreciated that these two fermented juices taste good, have a pleasant, refined aroma, and can be used as salad dressing
— in the case of fermented TM (tomato) juice, the best variant from the organoleptic point of view was the one fermented with the 26.1 strain; the taste was likened to green and the smell to that of a sour fruit (such as vax cherry), in a pleasant, appreciative way.

## 4. Conclusions

Lactic acid bacteria are widely used in the fermentation processes (animal and plant products). Due to the increasing interest in plant-based diets and the health advantages of consuming fermented beverages, there is an increasing attention on the selection of plant products and microorganisms used for the fermentation process.

Antibiotic resistance to probiotic LAB strains is a study that needs to be further explored because it represents a risk factor for human health, as this resistance can also be transferred to pathogenic species. As perspectives, the strains from this study showing antibiotic sensitivity, the presence of several functional genes (pH survival, starch metabolism, folate synthesis, and riboflavin synthesis) together with the production of organic acids, can be considered functional and used in future experiments to confirm the probiotic character. Using controlled fermentation with selected LAB strains can increase the quality of fermented vegetable juices. Further research studies should focus on the sensory analysis of the fermented vegetable juices with expert panels, because these types of functional beverages represent an interesting, relevant, and healthy alternative for the consumers. These vegetables fermented beverages will offer the pleasure of improved flavored drinks with organoleptic changes (compared with unfermented variants), the benefits of microbial composition (strengthening defense against pathogens, bioavailability of useful compounds) and a cost-effective alternative.

## Figures and Tables

**Table 1:** Functional LAB strains used in this study.

Species	Strain Designation (Collection Number)	Isolation Source	Reference
*Lactococcus lactis*	19.3 (R19316) ^b^	Milk	[24]
*Lactobacillus acidophilus*	IBB (ICCF 416) ^a^	Yogurt	[29]
*Lactobacillus amylolyticus*	P40 (OP524193) ^c^	Bors	[24]
	P50 (OP524194) ^c^		
*Lactobacillus helveticus*	34.9 (R19426) ^b^	Fermented milk	[30]
*Lactobacillus plantarum*	BR9	Braga	[28]
	CR1	Water kefir	
	26.1	Fermented milk	[24]
	L26	Bors	[25]
	L35		
	L22		
	L61	Pickled cabbage	
	L58		
*Streptococcus thermophilus*	ST111	Yogurt	[31]
*Leuconostoc mesenteroides*	21.2 (R24209) ^b^	Milk	[32]
	P109	Pepper	[33]
	P124 (OP546102) ^c^	Beans	
*Leuconostoc citreum*	Fv177	Pickles	[34]
	Fv52		

^a^ Romanian Collection of Industrial Microorganisms—CMII-ICCF-WFCC232. ^b^ R = Research Collection, Laboratory of Microbiology, Ghent University, Belgium. ^c^ https://www.ncbi.nlm.nih.gov/genbank/ (accessed on 11 August 2022).

**Table 2:** List of antibiotics used in the study.

Antibiotic	Concentration /Disc	Code	Producer	Class	Target
Ampicillin	10 µg	AMP 10	RightChoice Diagnostics, Yavne 70650, Israel	Β-Lactam	Cell wall
Chloramphenicol	30 µg	C 30	Liofilchem^®^, 64026 Roseto degli Abruzzi (TE), Italy	Amphenicol	Protein synthesis, 50S
Erythromycin	15 µg	E15	Liofilchem^®^, 64026 Roseto degli Abruzzi (TE), Italy	Macrolide	Protein synthesis, 50S
Gentamicin	30 µg	CN 30	Liofilchem^®^, 64026 Roseto degli Abruzzi (TE), Italy	Aminoglycoside	Protein synthesis, 30S
Kanamycin	30 µg	K 30	Liofilchem^®^, 64026 Roseto degli Abruzzi (TE), Italy	Aminoglycoside	Protein synthesis, 30S
Rifampicin	30 µg	RD 30	Liofilchem^®^, 64026 Roseto degli Abruzzi (TE), Italy	Rifamycin	RNA polymerase
Streptomycin	300 µg	S 300	Liofilchem^®^, 64026 Roseto degli Abruzzi (TE), Italy	Aminoglycoside	Protein synthesis, 30S
Tetracycline	30 µg	TE30	Liofilchem^®^, 64026 Roseto degli Abruzzi (TE), Italy	Tetracycline	Protein synthesis, 30S

**Table 3:** Primers for antibiotic resistance genes.

Antibiotic	Antibiotic Resistance Gene	Primer Sequences (5′–3′)	Amplicon Size (bp)	Annealing T (°C)	References
Ampicillin	*blaZ*	Fw: ACTTCAACACCTGCTGCTTTC Rev: TAGGTTCAGATTGGCCCTTAG	240 pb	61 °C	[36]
	*bla*	Fw: CATARTTCCGATAATASMGCC Rev: CGTSTTTAACTAAGTATSGY	297 pb	60 °C	
Chloramphenicol	*catA*	Fw: GGATATGAAATTTATCCCTC Rev: CAATCATCTACCCTATGAAT	486 pb	60 °C	[35]
Erythromycin	*erm(B)-1*	Fw: CATTTAACGACGAAACTGGC Rev: GGAACATCTGTGGTATGGCG	405 pb	54 °C	[37]
Gentamicin	*aac(6′)-aph(2″)*	Fw: CCAAGAGCAATAAGGGCATA Rev: CACTATCATAACCACTACCG	220 pb	60 °C	[35]
Kanamycin	*aph(3′)-IIIa*	Fw: GCCGATGTGGATTGCGAAAA Rev: GCTTGATCCCCAGTAAGTCA	292 pb	52 °C	
Streptomycin	*ant(6)*	Fw: ACTGGCTTAATCAATTTGGG Rev: GCCTTTCCGCCACCTCACCG	597 pb	61 °C	
Tetracycline	*tet(M)*	Fw: GGTGAACATCATAGACACGC Rev: CTTGTTCGAGTTCCAATGC	401 pb	52 °C	[37]

**Table 4:** Primers used for functional gene detection [38].

Functional Feature	Primer	Synthesis Product	Nucleotide Sequence 5′–3′	Annealing Temperature	Amplicon Size
pH Survival	LBA1272	Cyclopropane FA synthase	Fw: GGCCGGTGTTCCACTAGTCC Rev: ACGTTGGGTCGATTTGACGA	60 °C	203 bp
	dltD	D-alanine transfer protein	Fw: TTCGCCTGTTCAAGCCACAT Rev: ACGTGCCCTTCTTTGGTTCC		283 bp
Folate synthesis	folP	Dihydropteroate synthase/dihydropteroate pyrophosphorylase	Fw: CCASGRCSGCTTGCATGAC Rev: TKACGCCGGACTCCTTTTWY	61 °C	261 bp
	folK	2-amino-4-hydroxy-6-hydroxymethyldihydropteridine diphosphokinase	Fw: CCATTTCCAGGTGGGGAATC Rev: GGGGTGGTCCAGCAAACTT		214 bp
Starch metabolism	agl	α-glucosidase	Fw: GCSAAAATGCTAGCGACYMT Rev: CCACTGCATYGGYGTACGY		236 bp
	α-amy	α-amylase	Fw: AGATCAGGCGCAAGTTCAGT Rev: TTTTATGGGCACACCACTCA	62 °C	220 bp
	malL	Oligo-1,6-glucosidase	Fw: TTGCCTAACAACTGGGGTTC Rev: ATCAACGCCTTTGTTCAACC		177 bp
Riboflavin synthesis	ribA	3,4-dihydroxy-2-butanone 4-phosphate synthase/GTP cyclohydrolase II	Fw: TTTACGGGCGATGTTTTAGG Rev: CGACCCTCTTGCCGTAAATA		121 bp

**Table 5:** Vegetable juices used in this study for LAB fermentation.

Raw Material	Preparation	Juice Code
Beet	Freshly squeezed beetroot juice, autoclaved at 121 °C for 15 min	SF
Carrots, celery, and beets	Mixture of freshly squeezed juice from carrot, celery, and beet roots, in 2:1:2 (*v/v/v*) ratio, autoclaved at 121 °C for 15 min	MTS
Tomato juice	Fine blended tomato juice, autoclaved at 121 °C for 15 min	TM

**Table 6:** Antibiotic susceptibility of the studied LAB strains.

Strain	Amp10	C30	CN30	E15	K30	RD30	S300	TE30
**L22**	R	S	S	S	T	S	S	S
**L26**	T	S	S	S	T	S	S	T
**L35**	R	T	T	S	T	S	T	T
**L58**	T	T	S	T	T	S	T	T
**L61**	R	S	S	S	T	S	T	T
**19.3**	R	S	S	S	S	S	S	S
**21.2**	R	T	T	T	R	S	T	T
**26.1**	R	S	S	S	T	S	T	T
**34.9**	R	S	S	S	T	S	S	S
**P40**	R	S	S	S	R	S	S	S
**P50**	R	T	S	T	R	S	T	S
**P109**	R	S	S	S	R	T	T	T
**P124**	R	T	S	S	R	S	T	T
**Fv52**	R	S	S	S	T	S	S	S
**Fv177**	R	S	T	S	R	S	T	T
**BR9**	T	S	S	S	T	S	S	T
**CR1**	T	S	S	S	T	S	S	T
**IBB**	R	S	S	T	T	S	S	S
**ST111**	R	S	S	T	S	S	S	S

Where: S—antibiotic sensitive strain, T—antibiotic tolerant strain, susceptible to develop antibiotic resistance, R—antibiotic resistant strain at the tested concentration.

**Table 7:** Antibiotic resistance character in the analyzed LAB strains.

Strain	Main Phenotype	Antibiotic Resistance Genes Detected by Multiplex PCR
**L22**	Amp^R^	-
**L26**	Amp^T^, Kan^T^	*bla*
**L35**	Amp^R^	-
**LAB43**	-	-
**L58**	Amp^T^, Chl^T^, Ery^T^, Kan^T^, S^T^	*bla, ant(6)*
**L61**	Amp^R^	-
**BR9**	Amp^T^, Kan^T^	*bla, aph(3″)III*
**CR1**	Amp^T^, Kan^T^	*aph(3″)III*
**Fv52**	Amp^R^	*blaZ*
**Fv177**	Amp^R^, Kan^R^	-
**P124**	-	*aph(3″)III*
**ST111**	Amp^R^	*blaZ*
**26.1**	Amp^R^, Kan^R^	*blaZ, bla, aph(3″)III*
**34.9**	Amp^R^	*blaZ*

Where R—resistant, T—tolerant.

**Table 8:** Functional genes in the analyzed LAB strains.

Strain/Gene	*LBA1272*	*dltD*	*folP*	*folK*	*agl*	*α-amy*	*malL*	*ribA*
L22	+	−	−	−	−	−	−	−
L26	+	−	+	−	+	+	−	+
L35	+	−	+	−	+	+	−	+
LAB43	+	+	+	−	+	+	−	+
L58	+	−	+	−	+	+	+	+
L61	−	−	+	−	+	+	+	+
BR9	+	−	+	−	+	+	+	+
CR1	+	−	+	−	+	+	+	+
Fv52	+	−	+	−	+	+	+	+
Fv177	+	−	+	−	+	+	+	+
P124	+	−	+	−	+	+	+	+
ST111	+	−	+	−	+	+	+	+
26.1	+	−	+	−	+	+	+	+
34.9	+	−	+	−	+	+	+	+

Where + = the presence of gene specific amplicon; − = lack of specific amplification product.
